# Punching Shear Behavior of Two-Way Concrete Slabs Reinforced with Glass-Fiber-Reinforced Polymer (GFRP) Bars

**DOI:** 10.3390/polym10080893

**Published:** 2018-08-09

**Authors:** Minkwan Ju, Kyoungsoo Park, Cheolwoo Park

**Affiliations:** 1 Department of Civil and Environmental Engineering, Yonsei University, 50 Yonsei-ro, Seodaemun-gu, Seoul 03722, Korea; j_dean21@naver.com (M.J.); k-park@yonsei.ac.kr (K.P.); 2 Department of Civil Engineering, Kangwon National University, 346 Joongang-ro, Samcheok, Kangwon 25913, Korea

**Keywords:** two-way concrete slabs, GFRP bar, equivalent reinforcement ratio, punching shear strength

## Abstract

This study investigated the punching shear behavior of full-scale, two-way concrete slabs reinforced with glass fiber reinforced polymer (GFRP) bars, which are known as noncorrosive reinforcement. The relatively low modulus of elasticity of GFRP bars affects the large deflection of flexural members, however, applying these to two-way concrete slabs can compensate the weakness of the flexural stiffness due to an arching action with supporting girders. The test results demonstrated that the two-way concrete slabs with GFRP bars satisfied the allowable deflection and crack width under the service load specified by the design specification even in the state of the minimum reinforcement ratio. Previous predicting equations and design equations largely overestimated the measured punching shear strength when the slab was supported by reinforced concrete (RC) girders. The strength difference can be explained by the fact that the flexural behavior of the supporting RC beam girders reduces the punching shear strength because of the additional deflection of RC beam girders. Therefore, for more realistic estimations of the punching shear strength of two-way concrete slabs with GFRP bars, the boundary conditions of the concrete slabs should be carefully considered. This is because the stiffness degradation of supporting RC beam girders may influence the punching shear strength.

## 1. Introduction

Steel bar corrosion in reinforced concrete (RC) structures adversely impact on the durability and structural capacity of RC members. The consistently increasing maintenance costs of repairing and strengthening RC structures has led owners to seek more efficient and affordable solutions through the use of FRP bars [1]. FRP bars have been widely considered as substitutes for the reinforcement of steel bars in previous RC structures due to advantages such as their high resistance to electrochemical corrosion, high strength-weight ratio, and lightness [2,3,4,5]. However, rather than their strength, the lower flexural stiffness of FRP bars, compared to steel bars, is a more significant problem with respect to serviceability in terms of deflection and crack width [6]. There have been efforts to investigate the structural capacity of two-way concrete slabs reinforced with FRP bars. Full-scale, two-way concrete slabs characterized by structural variables such as the compressive strength of concrete, reinforcement ratio, and the thickness of the slab have been tested and their behavior has been studied [7,8]. Two-way concrete slabs can be considered a good application for FRP bars to overcome the lack of flexural stiffness as a result of compressive membrane action, which is similar to the arching action. A previous study reported that when a two-way concrete slab is restrained at the edges, it may not require flexural reinforcement to resist wheel loads; accordingly, for serviceability reasons, the minimum reinforcement was discussed [9]. This arching action effect has been implemented in some design specifications [10,11]. Based on the demonstration of the arching action effect, some innovative research on steel-free deck slabs has been proposed [12,13,14]. For the punching shear strength of FRP-reinforced concrete slabs, a comparative study was conducted with various prediction models. It was found that the equivalent steel reinforcement ratio was a better approach for more accurate prediction of the punching shear strength [15].

This study investigates the structural performance of full-scale, two-way concrete slabs reinforced with glass-fiber-reinforced polymer (GFRP) bars. Slab specimens were restrained by two RC beam girders with hinged supports. The reinforcing types considered were steel bars and GFRP bars of two different diameters, which include the minimum reinforcement ratio of 0.002 recommended by the specifications of the Canadian Standards Association (CSA, Mississauga, ON, Canada, 2000) [16]. The structural performance was evaluated with respect to the strength and serviceability. For the strength, the applied load and deflection relationship was measured and the load carrying capacity at the specific design strength was investigated. For the serviceability, the deflection and crack width were measured and the allowance in structural design was discussed at the service load state. The punching shear strength was calculated using the three equations from American Concrete Institute (ACI) [17], CSA [18], Menétrey [19] and compared with the experimental results. Accordingly, the reduction of the tested punching shear strength by the supporting RC beam girders is discussed.

## 2. Experimental Program

### 2.1. Materials

The two-way concrete slabs were fabricated using normal weight and ready-mixed concrete, which was 30 MPa of the designed compressive strength. The average compressive strength of five cylindrical concrete specimens (∅100 mm × 200 mm) was obtained as 36.7 ± 1.2 MPa. The GFRP bars used in this study were manufactured by the typical pultrusion process by braiding the fiber ribs on the surface of the GFRP bar. The GFRP bars were made of polyvinyl alcohol (PVA) resin reinforced with E-glass fiber, with a fiber volume fraction of 65% by weight. The nominal diameter of D13 for the steel bar, and D16 and D19 for the FRP bars were 12.7, 16.1 and 19.1 mm, respectively.

The nominal diameters were obtained by an immersion test based on density. The tensile properties of the GFRP bars were determined using the tensile test in compliance with ACI 440 3R-04 [20]. Table 1 shows the mechanical properties of the reinforcement used in this study.

### 2.2. Test Specimens

**Figure 1:**
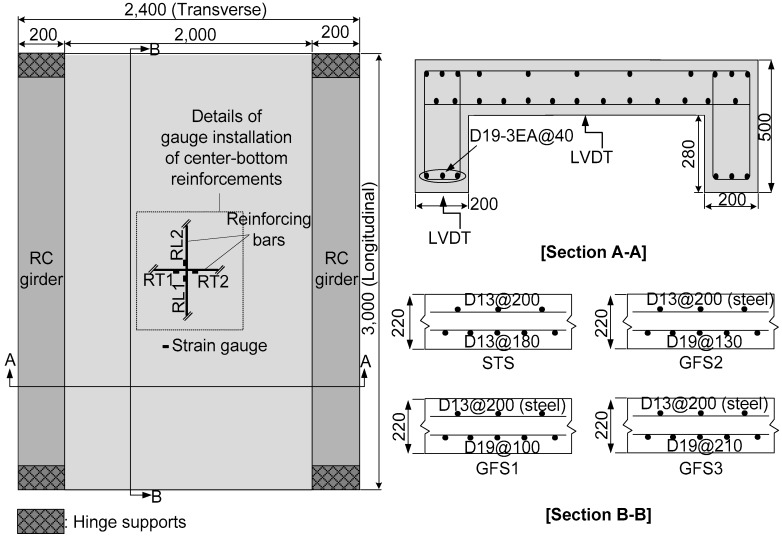
Specimen details of the full-scale, two-way concrete slab specimens (unit in mm).

A total number of four full-scale two-way concrete slabs were fabricated with two supporting RC beam girders reinforced with steel reinforcements. At 2400 mm wide and 3000 mm long, the considered thickness of 220 mm satisfies the minimum depth calculated using the formula 1.2 (S + 3000)/30 [22], where *S* is the center-to-center spacing of the supports. The recommended clear cover of the concrete bridge deck was 25.4 mm [23]; however, in this study, the clear cover depth was determined as 30 mm, which satisfies the minimum cover thickness recommended by the specifications. Figure 1 shows the geometry of the test specimens. The top bars were designed with steel reinforcements not considered the test variable in this study. Table 2 summarizes the reinforcement details of the two-way concrete slabs. The specimen ID was classified as a steel bar slab (STS) specimen for the two-way concrete slabs reinforced with steel bars, and as a glass fiber bar slab (GFS) specimen for the two-way concrete slabs reinforced with GFRP bars.

Each specimen was designed employing the equivalent reinforcement ratio of *ρ*_eq_ (=*ρ*_frp_ (*E*_frp_/*E*_s_)) along the main transverse direction at the bottom of the specimen. In order to compare with the STS specimen, the GFS1 specimens were designed using the equivalent reinforcement ratio of 0.36, similar to the reinforcement ratio of 0.39 for the STS specimen. It was expected that the flexural stiffness would be nearly the same but with different ultimate strengths. The GFS1 and GFS2 specimens were employed for the evaluation of the difference in the structural behavior near the minimum reinforcement ratio of 0.002. CSA (2012) provided a reinforcement ratio for the longitudinal reinforcement of the deck slab as a function of the effective spacing between the girders and not exceeding 67% of the transverse reinforcement. Hinges installed at the end of the two girders allowed the supporting RC beam girders to be rotated to the longitudinal direction. This design concept for the girders was intended to impose an additional flexural effect on the two-way concrete slabs so that the ultimate punching shear strength may be reduced compared to the slabs supported by the high stiffened girders, which are hardly able to deflect.

### 2.3. Test Setup and Data Acquisition

**Figure 2:**
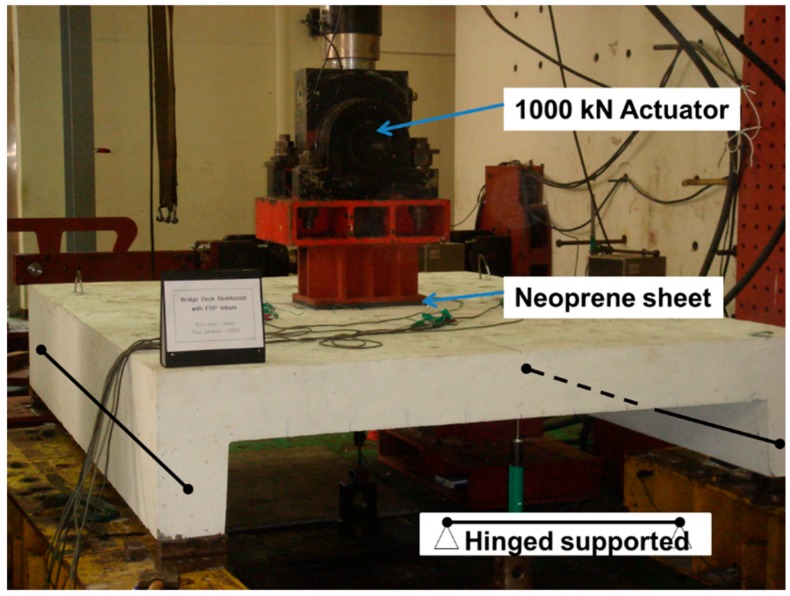
Experimental setup.

The concentrated load was applied at the center of the top surface of the test specimens with a 300 mm × 500 mm loading plate on a 30-mm thick neoprene sheet, to avoid the stress concentration at the edge of the loading plate. The MTS (Material testing systems, Eden Prairie, MN, USA) loading actuator was used with the capacity of 1000 kN, and the rate of loading was 1 mm/min. Two linear variable displacement transducers (LVDTs) with a measuring limit of a 100 mm stroke were also used to measure the vertical deflection at the bottom center of the slab and at the mid-span of the RC beam girders. During the loading test, the maximum crack width was measured using a crack width ruler at each designated step. The detailed test setup is shown in Figure 2.

## 3. Test Results and Discussions

### 3.1. Punching Shear Failure and Cracking Patterns

**Figure 3:**
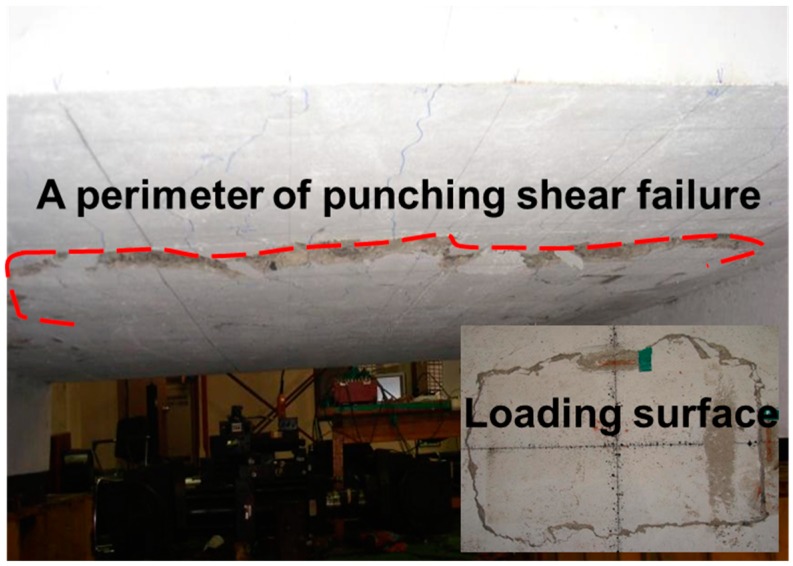
Punching shear failure at the bottom surface.

**Figure 4:**
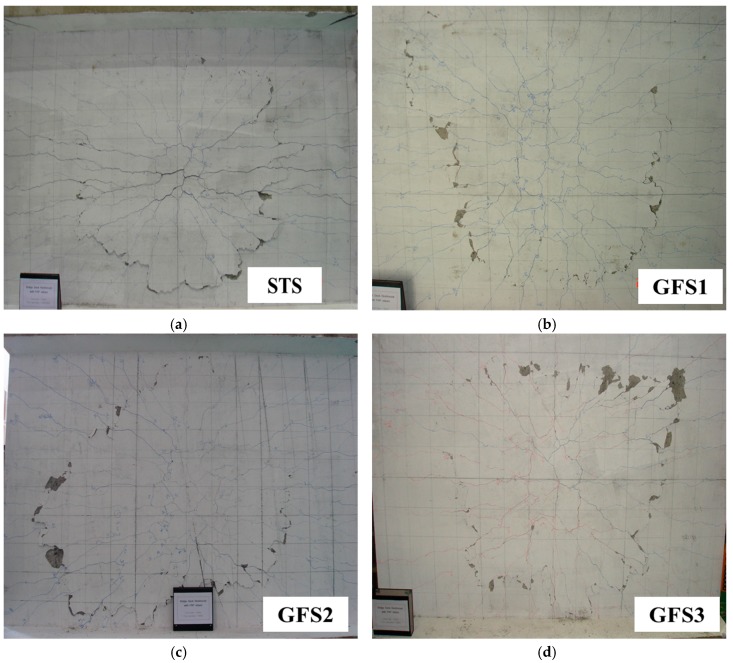
Punching shear failure at the bottom surface. (**a**) STS specimen; (**b**) GFS1 specimen; (**c**) GFS2 specimen; (**d**) GFS3 specimen.

The specimens showed that punching shear failure occurred underneath the loading point at the ultimate state. Brittle failure occurred and the loading top and bottom surface sank, as shown in Figure 3. The shear resistance of the reinforcements was controlled to prevent the collapse of the concrete due to punching shear failure. Figure 4 exhibits the cracking patterns of the punching shear failure. Many radial cracks were developed, including a few longitudinal cracks. For the GFS specimens, the transverse surface of punching shear failure was approximately closer to the inner edge of the supporting RC beam girders than the transverse surface of the STS specimen. The reason for this may be the relatively low flexural stiffness, even though the equivalent reinforcing ratio by modulus ratio was appropriately designed. Another reason may be that the bond capacity between the concrete and GFRP bars in the transversal direction was not the same as that obtained with steel bars. The difference in the punching shear failure area between the top and bottom of the test specimen induces the punching cone angles. The angles were calculated using the relationship between the slab thickness and the projective distance from the loading plate to the punching shear.

**Figure 5:**
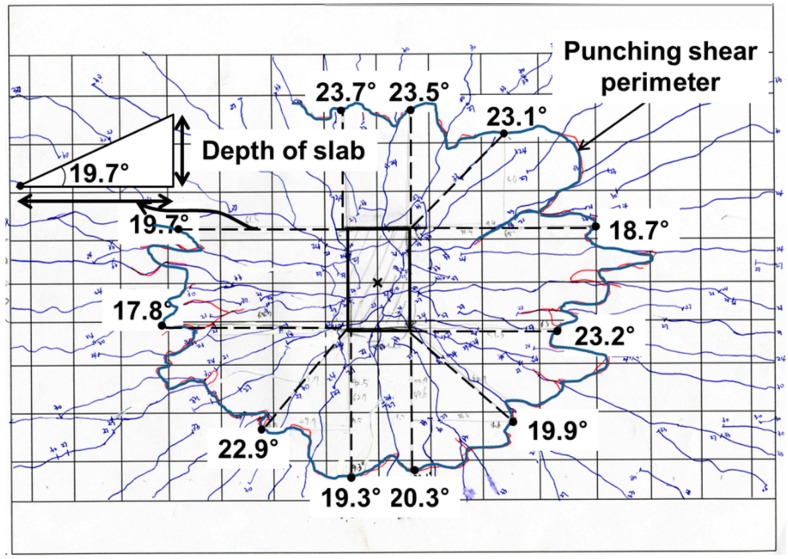
Illustration of the measured punching cone angle (STS).

Figure 5 shows an example of the punching shear surface with the measured punching cone angles for the STS specimen. The measurement points of the angles were determined using the point of intersection of the punching shear failure and the extended line from the four edges with the vertical, horizontal, and 45° direction, respectively. The measured and average punching cone angles are listed in Table 3. The average angle of the STS specimen was calculated as 21.1°, and for the GFS1, GFS2, and GFS3 specimens they were calculated as 22.8°, 21.3° and 22.8°, respectively. The average difference of the measured angles was small. For the standard deviation, however, the GFS specimens exhibited almost twice the deviation of the STS specimen. This is because the GFS specimen had wider punching shear failure, which may make it biased compared to the supporting RC beam girders, which can be explained by the fact that GFS specimens may be even more affected by the flexure of the supporting RC beam girders, even though they were normally designed using the equivalent reinforcing ratio of GFRP bars.

### 3.2. Load and Deflection Behavior

**Figure 6:**
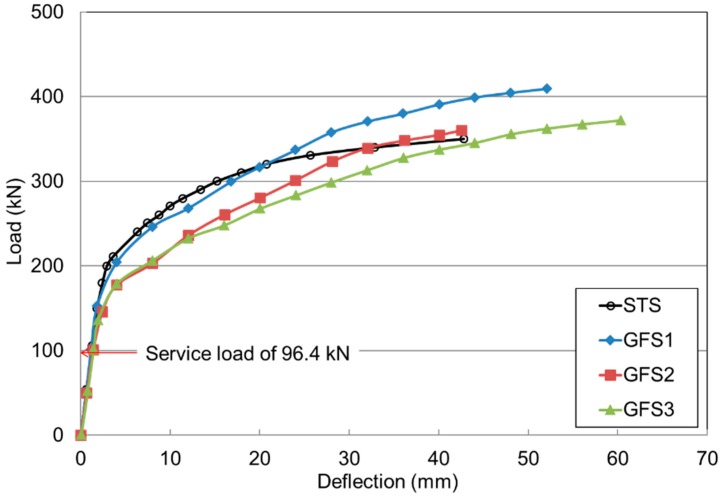
Load and deflection relationship of GFS specimens.

Figure 6 illustrates the load and deflection relationship at the bottom center of the slabs. The STS and GFS1 specimens exhibited similar load and deflection relationships until the elastic range of approximately 300 kN. The STS and GFS1 specimens had similar elastic behavior before the supporting RC beam girders yielded at approximately 230 kN. As expected, the GFS2 and GFS3 specimens showed lower punching shear strength than the GFS1 specimen because of the lower reinforcement ratio. For the load carrying capacity, the GFS1 specimen was 20% higher and the deflection was approximately twice as large as the deflection of the STS specimen. The GFS2 and GFS3 specimens had an ultimate strength similar to the ultimate strength of the STS specimen, where a larger deflection and lower stiffness behavior occurred. It was evaluated that the tensile strength and strain of the GFRP bars were higher than those of the steel bars. The effect on the load and deflection relationship of reducing the reinforcement ratio to the minimum reinforcement ratio of 0.002 was investigated. In accordance with American Association of State Highway and Transportation Officials (AASHTO) (2007), the service load, *P*_ser_, for the specimens was calculated as 1.33 × 72.5 = 96.4 kN (72.5 kN was the rear wheel axis load of the design truck, and the factor 1.33 was the dynamic load allowance of 33%). For the design factored load, *P*_f_ was taken as 1.33 × 1.8 × 72.5 = 173.6 kN, where the factor 1.8 was the live load combination factor specified by the ultimate level I. Compared to the design service load calculated as 96.4 kN, it was evaluated that the tested initial cracking load was higher. For the design purpose, the deflection allowance was specified as span/800 (=2.75 mm) for the vehicle load in general by AASHTO (2007), which was specified in AASHTO (2009) [24] as the applicable provision. The maximum deflections measured at the design service loads were 1.27, 1.14, 1.40 and 1.42 mm, for STS, GFS1, GFS2, GFS3, respectively. Note that the GFS3 specimen was designed using the minimum reinforcement. Thus, the test results indicated that even applying the minimum reinforcement ratio of the FRP bar is applicable for the serviceability and deflection under the designed service load.

Table 4 summarizes the results of the strength capacity for the initial cracking loads (*P*_cr_), ultimate loads (*P*_u_) and strength ratios for service (*P*_ser_) and factored loads (*P*_f_), respectively. It was evaluated that the strength ratio of the cracking load to service load ranged from 1.4 to 1.6; and the ratio of *P*_u_ to *P*_f_ ranged from 2.1 to 2.4. This implies that the strength capacity of the two-way concrete slabs reinforced with GFRP bars was sufficient to resist the designed service and factored loads under punching shear stress, even though they were reinforced using the minimum reinforcement ratio of the GFRP bars.

### 3.3. Load and Strain Behavior

**Figure 7:**
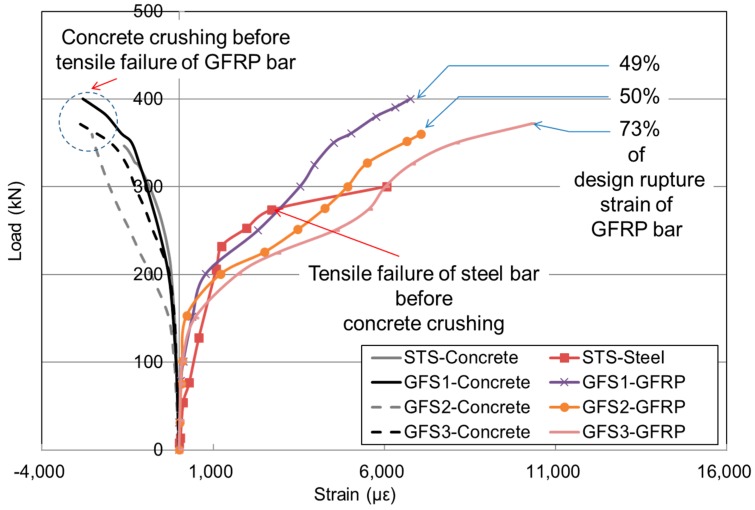
Load and strain relationship of the STS and GFS specimens.

Figure 7 shows the load and strain relationship of the concrete and reinforcement (bottom transverse). Prior to the application of the cracking load listed in Table 4, all the two-way concrete slabs exhibited a similar stiffness capacity; however, they began to exhibit different trends in strain after cracks occurred for the reinforced ones. As the reinforcement ratio reached the minimum reinforcement ratio for GFS specimens, an increment in tensile strain varied largely as intended. The noticeable differences between the STS and GFS specimens were in their modes of failure. The STS specimen failed after the steel bar yielded, whereas the GFS specimen failed with concrete crushing failure before the FRP bar had reached the ultimate strain. The strains of GFRP bars at failure were 43–73% of the designed rupture strain. The result implies that the structural capacity at the ultimate load was validated even for the minimum reinforcement ratio of the GFRP bars caused by a compensation effect on the flexural capacity through compression membrane action resulting from the edge restraints. ACI 440 1R-15 specifies the creep rupture stress limit of GFRP bars as 20% of the designed tensile stress. Based on this recommendation, the measured strain at the factored load was approximately 1000 με, which is less than 10% of the designed tensile strain. Thus, it was found that the load and strain behavior of the GFS series was stable even though the minimum reinforcement ratio of the GFRP bars was employed.

### 3.4. Load and Crack Width Behavior

FRP bars are a corrosion-free reinforcing material, and the maximum allowable crack width can be within a wider range, especially when the corrosion due to reinforcement is a primary reason for the allowable crack width limitations. Other considerations with regard to acceptable crack width limits include aesthetics and shear effects. Japan Society of Civil Engineering (JSCE) (1997) [25] only considers aesthetics in setting the maximum allowable crack width of 0.5 mm, and CSA (2006) implicitly allows crack widths of 0.5 mm when FRP bars are used for exterior exposure and 0.7 mm for interior exposure.

**Figure 8:**
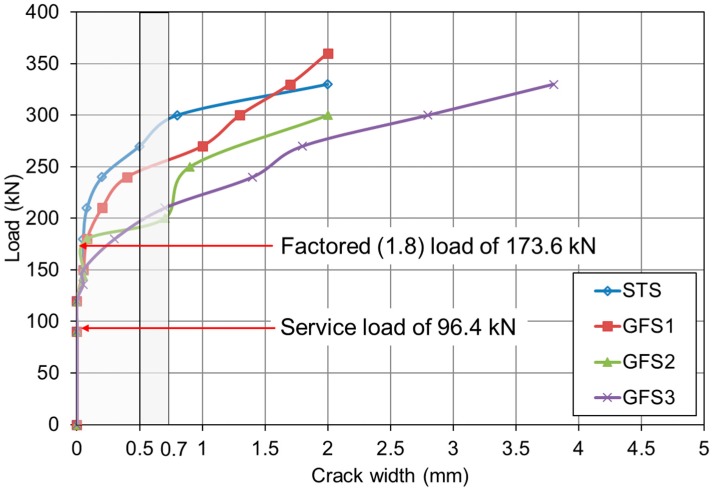
Measured load and crack width relationships of STS and GFS specimens.

Figure 8 shows the relationship of the measured load and crack widths. As can be seen from the graphs, all the test specimens exhibited crack widths that were less than the allowable 0.5 mm at the service load, *P*_ser_ = 96.4 kN. At the factored load of 173.6 kN with a live load factor of 1.8 for ultimate level I, based on the AASHTO (2007) recommendation, the STS specimen yielded the minimum crack opening width among all the slabs, while the GFS1 specimen, which had an equivalent reinforcement ratio, exhibited a larger crack width. For the entire GFS series, the crack width at *P*_f_ increased as the reinforcement ratio decreased; however, they were within the allowable 0.5-mm limit. Accordingly, it should be noted that the GFS series equally experienced flexure in the longitudinal direction caused by the supporting RC beam girders. Thus, the boundary conditions in this study may produce greater deflection and crack widths compared to the concrete slabs on the high stiffening steel girders. Despite the additional flexure of the supporting RC beam girders, the GFS series excellently maintained the allowable crack width for both the service load and factored load.

## 4. Strength Evaluation of Two-Way Concrete Slabs Reinforced with GFRP Bars in Punching Shear

**Figure 9:**
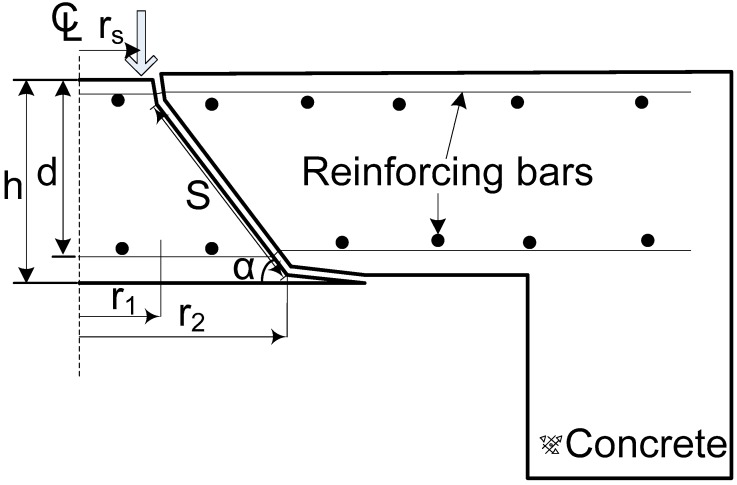
A scheme of the mechanism of punching shear failure.

To evaluate the punching shear strength, the equations of Menétrey, ACI 440 and CSA S806 were employed and the tested and predicted punching shear strength was compared. These equations are introduced below. Menétrey’s theory (2002), which includes the structural effect of the strength ratio, punching cone angle, and structural dimensions, was adapted in this study. The equation is shown in Equation (1). Figure 9 shows a scheme of the mechanism of punching shear failure.

(1) $$F_{\mathrm{Menétrey}}=F_{\mathrm{ct}}+F_{\mathrm{dowel}}+F_{\mathrm{sw}}+F_{p}$$

where,

- $F_{\mathrm{ct}}$ = the vertical component of the concrete tensile force obtained by integrating the vertical components of the tensile stresses in concrete
- $F_{\mathrm{dowel}}$ = the dowel force contribution of the flexural reinforcement
- $F_{\mathrm{sw}}$ = the vertical components of the forces in the studs
- $F_{p}$ = the effect of the prestressing tendon

The assumptions for the analysis of the GFRP-reinforced concrete slabs are explained as follows.

(1) The dowel action (*F*_dowel_) of the FRP bar is neglected due to the lower strength and stiffness of the FRP bar in the transverse direction (ACI 440 1R-15 2015).
(2) The effect of the studs (*F*_sw_) is replaced with stirrups crossing the punching cracks. This effect is neglected and discussed as the design safety in the analysis results.
(3) The tendon effect (*F*_p_) is not available in this experimental test.
(4) The load on the two-way concrete slabs is regarded as the tire contact load of vehicle and is practically considered as a concentrated load.

*F*_ct_ is the term of the concrete tensile force introduced in Equation (2). It is a function of the loading radius, effective depth of the two-way concrete slabs, concrete tensile stress, and three influencing constants from Figure 9.

(2) $$F_{\mathrm{ct}}=\pi \left(r_{1}+r_{2}\right)sf_{t}^{2/3}\xi \eta \mu$$

According to the theory, the concrete tensile force is determined by integrating the vertical component of the tensile stress around the punching crack. The punching crack is assumed to be a truncated cone shape comprised between two radii, *r*_1_ and *r*_2_. These radii are based on the specific two failure points as follows.

(3) $$r_{1}=r_{s}+\frac{1}{10}\frac{d}{tan\alpha }$$

(4) $$r_{2}=r_{s}+\frac{d}{tan\alpha }$$

where,

- $r_{s}$ = the radius of the column
- α = the punching cone angle
- d = the effective depth
- $f_{t}$ = the uniaxial tensile strength of concrete $=0.3f_{c}^{2/3}$

For the value of the radius of the column, the averaging radius of the loading plate was applied, obtained from the failure test. The angle was set for each two-way concrete slab from the experimental results for the punching shear failure surface. In this study, the average punching cone angle was used. The inclination length *s* is as follows.

(5) $$s=\sqrt{{\left(r_{s}-r_{1}\right)}^{2}+{\left(0.9d\right)}^{2}}$$

Influencing constants for Equation (2) are introduced in Equations (6)–(8). In the case of the applying reinforcement ratio, the reinforcement ratio (*ρ*) was replaced with the equivalent reinforcement ratio (*ρ*_eq_ = *ρ*_frp_ (*E*_frp_/*E*_s_))), including the modulus ratio of *E*_frp_*/E*_s_.

(6) $$\xi =\{\begin{array}{cc} -0.1\rho^{2}+0.46\rho +0.35 & \;0<\rho <2\%\\ 0.87 & \rho \geq 2\% \end{array}$$

(7) $$\mu =1.6{\left(1+\frac{d}{d_{a}}\right)}^{-1/2}$$

(8) $$\eta =\{\begin{array}{cc} 0.1{\left(\frac{r_{s}}{h}\right)}^{2}-0.5\left(\frac{r_{s}}{h}\right)+1.25 & \;0<\frac{r_{s}}{h}<2.5\%\\ 0.625 & \frac{r_{s}}{h}\geq 2.5\% \end{array}$$

where,

- ρ = the reinforcement ratio
- $d_{a}$ = the maximum aggregate size
- ξ, μ, η = influencing constants
- h = the slab thickness
- $E_{s}$ = the elasticity modulus of the steel bar
- $E_{\mathrm{frp}}$ = the elasticity modulus of the FRP bar

For the comparative study of the punching shear strength, the latest design specifications for FRP-bar-reinforced concrete structures such as ACI 440 1R-15 and CSA S806-12 were considered and are introduced below. The ACI 440 committee is currently considering the introduction of a new provision to account for the punching capacity of two-way concrete slabs reinforced with FRP bars in next edition, ACI 440.1R-15. This equation considers the effect of the reinforcement stiffness to account for the shear transfer in two-way concrete slabs. The modulus ratio for FRP and concrete was considered in Equation (9). It was transferred to *F*_ACI4401R-15_

(9) $$F_{\text{ACI4401R-15}}=\frac{4}{5}\sqrt{{f_{c}}^{\prime }}u_{0.5d}c$$

where,

- ${f_{c}}^{\prime }$ = specified compressive strength of concrete (MPa)
- $u_{0.5d}$ = control perimeter of the effective depth (mm)
- c=kd (neutral axis depth of the cracked transformed section, mm)
- $k=\sqrt{2\rho_{\mathrm{frp}}n_{\mathrm{frp}}+{\left(\rho_{\mathrm{frp}}n_{\mathrm{frp}}\right)}^{2}}-\rho_{\mathrm{frp}}n_{\mathrm{frp}}$
- $n_{\mathrm{frp}}=\frac{E_{\mathrm{frp}}}{E_{c}}$
- $\rho_{\mathrm{frp}}$ = reinforcement ratio of FRP bar
- $E_{\mathrm{frp}}$ = elasticity modulus of the FRP bar (MPa)
- $E_{c}$ = elasticity modulus of the concrete (MPa)

The Canadian Standard “Design and construction of building structures with fibre reinforced polymers”, CSA-S806-12, specifies the punching shear strength of FRP-reinforced concrete. It can be noted that these equations are the modified forms of those specified in the CSA (2004) [26], to account for the FRP-reinforcing bar ratio. The factored punching shear strength is determined by the lowest of the following Equations (10)–(12).

(10) $$F_{\text{CSA S806-12}}=0.028\lambda \phi_{c}\left(1+\frac{2}{\beta_{c}}\right){\left(E_{\mathrm{frp}}\rho_{\mathrm{frp}}f_{c}^{\prime }\right)}^{1/3}u_{0.5d}d$$

(11) $$F_{\text{CSA S806-12}}=0.147\lambda \phi_{c}\left(\frac{\alpha_{s}d}{u_{0.5d}}+0.19\right){\left(E_{\mathrm{frp}}\rho_{\mathrm{frp}}f_{c}^{\prime }\right)}^{1/3}u_{0.5d}d$$

(12) $$F_{\text{CSA S806-12}}=0.056\lambda \phi_{c}{\left(E_{\mathrm{frp}}\rho_{\mathrm{frp}}f_{c}^{\prime }\right)}^{1/3}u_{0.5d}d$$

- $\beta_{s}$ = ratio of the long side to the short side of the concentrated load or loading patch
- λ = density factor (i.e., for normal density concrete this is equal to 1)
- $\phi_{c}$ = concrete resistance factor (if unfactored, use 1.0)
- $\alpha_{s}$ = factor to adjust the concrete shear strength for support dimensions, equal to 4 for interior columns, 3 for edge columns and 2 for corner columns

Table 5 summarizes the results of the tested and predicted punching shear strengths for the GFRP-reinforced concrete slabs. The three equations provided similar results for the punching shear strength. For the test and predicted ratios, they ranged from 63% to 76%. Thus, the three equations overestimated the punching shear strength. Overestimated predictions must be avoided due to the safety requirements of structural design. In this study, the overestimation can be attributed to the reduction in strength due to flexure of the supporting RC beam girders. Originally, the above equations were derived using a flat slab for which the boundary condition was restricted under flexure. If the RC beam girders are allowed to largely bend, then the girder behavior can reduce the concentrated load of the concrete slab due to the additionally-imposed moment action in the longitudinal direction. Accordingly, the tested punching shear strength is going to be relatively low compared with that obtained by the three equations. In contrast to RC beam girders, a previous study tested a concrete slab with steel girders, which had much higher longitudinal flexural stiffness [9]. The comparison specimens were 3000 mm long and 2500 mm wide thick and the slab thickness was 200 mm. The slabs were supported on two steel girders spaced at 2000 mm center to center. The results are summarized in Table 6. The punching shear strength was higher than that of the test specimens in Table 5. The result for G-150-N was 360 kN due to the short thickness of 150 mm. The load and deflection relationships are represented as the linear behavior up to failure. They were caused by there being no or little deflection effect of the steel girders in the longitudinal direction. As a result of comparison with the prediction equations, they were shown to be underestimated or close to 1.0.

**Figure 10:**
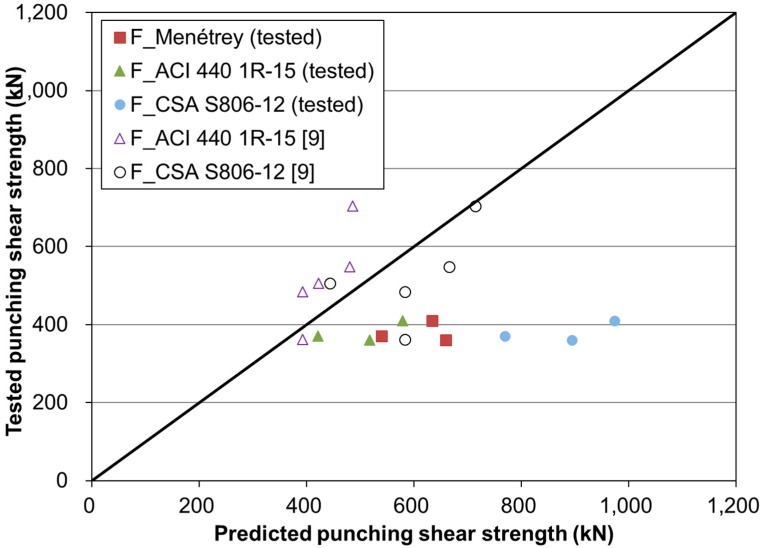
Results of tested and predicted punching shear strength.

From the comparison results as shown in Figure 10, the test specimens with RC beam girders, however, showed largely overestimated results. This difference must be caused by the flexural behavior of the girders. In particular, it is possible that the elasticity modulus of the RC beam girders is usually degraded due to corrosive conditions and repeated vehicle loads during their service life. Accordingly, the correlation between the flexural behavior of RC beam girders and the concentrated punching shear strength may be one of the significant issues in evaluating the serviceable or ultimate strength of concrete slabs reinforced with GFRP bars.

## 5. Conclusions

This study investigated the punching strength behavior of two-way concrete slabs reinforced with non-corrosive GFRP bars restrained by two RC beam girders. The punching shear strength, deflection and crack width were examined in terms of strength and serviceability performance. The punching shear strength was evaluated using the three equations, and the overestimation of the evaluated strength was discussed. The main conclusions are summarized as follows.

(1) All test specimens displayed punching shear failure. The STS specimen exhibited stiffer behavior until failure than the GFS specimens that were reinforced using an equivalent reinforcement ratio. For the tested service load of the GFS specimens, the load carrying capacity was 1.4–1.6 times higher than the designed service load. Additionally, the experimental results demonstrated that the structural capacity was sufficient to resist the concentrated design load due to the vehicle load even though the amount of GFRP bars was designed using the minimum reinforcement ratio.
(2) In comparison with the allowable deflection at the designed service load, the deflection of the GFS3 specimen, which was designed using the minimum reinforcement ratio, was measured as 1.42 mm. This was adequate as an allowable deflection. In comparison to the allowable crack width of 0.5 mm, the crack widths of the GFS specimens were lower than the allowable crack width (i.e., 0.5 mm). However, the allowable crack width of GFRP was larger than the allowable crack width of the RC member (i.e., 0.3 mm). The recommended allowable crack width must be well within the acceptable crack-width range for two-way concrete slabs reinforced with FRP bars, with higher strength and stronger durability under marine and ocean environments.
(3) The tested punching shear strength was compared with the predicted equations. It indicated that the predicted strength largely overestimated the experimental punching shear strength as compared with previous test results, which had a similar geometry, size and structural design process but highly stiffened girders. This can be explained by the effect of flexure on the supporting RC beam girders, which may be a significant parameter in the evaluation or design of the punching shear capacity of full-scale, two-way concrete slabs reinforced with GFRP bars. During their service life, this can be accelerated due to repeated vehicle loading on slabs. Accordingly, further investigations should be conducted in relation to the correlation between the punching shear strength and the stiffness degradation of supporting RC beam girders.

## Figures and Tables

**Table 1:** Mechanical properties of reinforcements.

	Bar ID *	Cross-Sectional Area (mm^2^)	Modulus of Elasticity (GPa)	Yield Strength (MPa)	Design Tensile Strength (MPa)	Design Rupture Strain (%)
Steel bars	D13	126.7	200	300	-	0.15
GFRP bars	D16 D19	203.6 286.5	47.8 46.7	-	691.0 687.0	1.4

* Provided by manufacturer (Dongwon Construction, Seoul, Korea, 2004) [21].

**Table 2:** Reinforcement details of the two-way concrete slab specimens.

Specimen ID	Bar Types	Transverse Direction		Longitudinal Direction		Reinforcement Ratio (%) *	
		Bottom	Top	Bottom	Top	*ρ*	*ρ*_eq_
STS	Steel	D13@180 mm	D13@200 mm (Steel)	D9@150 mm	D13@200 mm (Steel)	0.39	0.39
GFS1	GFRP	D19@100 mm		D19@140 mm		1.57	0.36
GFS2		D19@130 mm		D16@130 mm		1.20	0.28
GFS3		D19@210 mm		D16@210 mm		0.79	0.18

* For the transverse direction, the structural behavior is governed by the external load.

**Table 3:** Measured and average punching cone angle (unit in degrees).

Specimen ID	Max	Min	Average
STS	23.7	17.8	21.1 ± 2.1
GFS1	28.8	15.4	22.8 ± 4.0
GFS2	29.3	13.4	21.3 ± 4.2
GFS3	32.5	17.5	22.8 ± 4.9

**Table 4:** Summary of test results.

	Initial Cracking Load, *P*_cr_, (kN)	*P*_u_ (kN)	*P*_cr_/*P*_ser_	*P*_u_/*P*_f_
STS	150	355	1.6	2.0
GFS1	150	410	1.6	2.4
GFS2	144	360	1.5	2.1
GFS3	136	370	1.4	2.1

**Table 5:** Prediction and comparison results of tested punching shear strength.

Specimens	*F*_u_	*F* ^1^_Menétrey_	*F* ^2^_ACI4401R-15_	*F* ^3^_CSA S806-12_	*F*_u_/*F*^1^	*F*_u_ */F* ^2^	*F*_u_/*F*^3^
STS	355	733.5	-	-	0.48	-	-
GFS1	410	634.1	579.1	632.4	0.65	0.71	0.65
GFS2	360	659.4	517	581	0.55	0.70	0.62
GFS3	370	539.2	421.4	500	0.69	0.88	0.74
Average *					0.63	0.76	0.67

* Average for GFS specimens.

**Table 6:** Prediction and comparison results of previously tested specimens [9].

Specimens	*F*_u_ [9]	*F* ^2^_ACI4401R-15_	*F* ^3^_CSA S806-12_	*F*_u_/*F*^2^	*F*_u_/*F*^3^
C-175-N	530	-	-	-	-
G-175-N	484	392	582.4	1.23	0.83
G-150-N	362	392	582.4	0.92	0.62
G-175-H	704	485	713.7	1.45	0.99
G-175-N-0.75	549	480	665.7	1.14	0.82
G-175-N-0.35	506	422	442.9	1.20	1.14
Average *				1.19	0.88

* Average for G specimens.
